# mTOR inhibitors, a new era for metastatic luminal HER2-negative breast cancer? A systematic review and a meta-analysis of randomized trials

**DOI:** 10.18632/oncotarget.7446

**Published:** 2016-02-17

**Authors:** Maria Saveria Rotundo, Teresa Galeano, Pierfrancesco Tassone, Pierosandro Tagliaferri

**Affiliations:** ^1^ Department of Experimental and Clinical Medicine, Medical Oncology, Magna Graecia University, Viale Europa, and Catanzaro, Italy; ^2^ Department of Experimental and Clinical Medicine, Translational Medical Oncology, Magna Graecia University, Viale Europa, Catanzaro, Italy; ^3^ Sbarro Institute for Cancer Research and Molecular Medicine, College of Science and Technology, Temple University, Philadelphia PA, USA

**Keywords:** metastatic breast cancer, luminal breast cancer, meta-analysis, hormonal therapy, mTOR inhibitor

## Abstract

We evaluated if standard hormonal therapy (HT) could be improved by the addition of mammalian target of rapamycin inhibitors (mTOR-I) in metastatic luminal breast cancer. A meta-analysis on 4 phase II-III randomized clinical trials was performed. Pooled hazard ratio (HR) for progression free survival (PFS)/ time to progression (TTP) was 0.62 in favor of mTOR-I+HT arm (95% confidence interval [CI] 0.55-0.70; *p*<0.0001). There was significant heterogeneity for PFS/TTP (Cochran's Q 32, *p*<0.0001, I^2^ index 90.6%). Pooled HR for overall survival (OS) was 0.84 in favor of the combination arm (95% CI 0.71-0.99; *p*=0.04). Heterogeneity was not significant (Cochran's Q 4.47, *p*=0.1, I^2^ index 55.3%). Pooled risk ratio (RR) for objective response rate (ORR) was 0.88 in favor of experimental arm (95% CI 0.85-0.91; *p*<0.0001). Heterogeneity was not significant (Cochran's Q 2.11, *p*=0.3, I^2^ index 5.2%). Adverse events (AEs), in particular those of grade 3-4, mostly occurred in mTOR-I+HT arm. Combination therapy of HT *plus* mTOR-I improves the outcome of metastatic luminal breast cancer patients. Our results provide evidence of a class-effect of these targeting molecules.

## INTRODUCTION

In the last decade the development of new therapeutic approaches for luminal breast cancer [1] has been an active area of investigation, since this molecular subtype could indeed represent a stand-alone class in breast cancer scenario [2]. In this context, specific targeting of mTOR, a molecular checkpoint involved in cell proliferation and immune microenvironment modulation, is a promising approach to improve the current treatment [3–4].

On this basis, we performed a systematic review on randomized trials investigating mTOR-I in combination to HT as compared to HT alone in metastatic luminal breast carcinoma. Subsequently, we conducted a meta-analysis to determine benefit and safety of the combined treatment. The aim of our study was to investigate if the addition of mTOR-I to standard HT could produce a class-effect on luminal breast cancer outcome.

## RESULTS

### Studies selection

**Figure 1:**
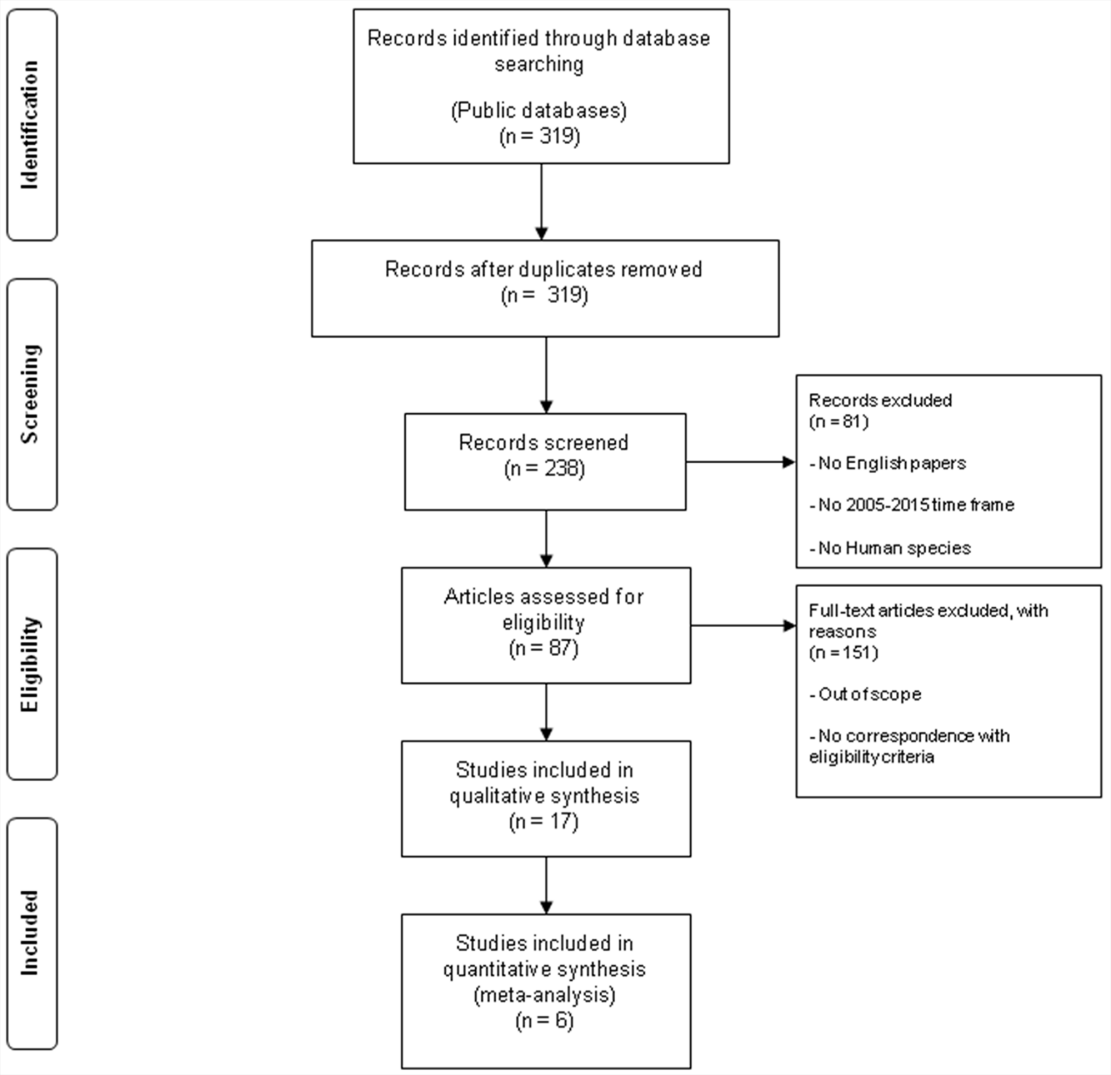
Flow chart of the literature search used in this meta-analysis Public databases (PubMed, Embase, Central Registry of Controlled Trials of the Cochrane Library) full texts and abstracts were performed to track relevant references for the 2005-2015 time frame.

Three-hundred nineteen articles from MEDLINE bibliographical database were found. All no comparative studies, no randomized clinical trials, studies that not involved our target drugs were excluded. The remaining 87 articles were further reviewed and only 6 articles met our inclusion criteria. The searching and selection process is outlined in Figure 1.

### Individual studies characteristics and results

All studies were conducted on postmenopausal women with hormone receptor positive and HER2-negative advanced breast cancer. One phase II trial evaluated everolimus *plus* tamoxifen *versus* tamoxifen alone (TAMRAD). One phase III trial evaluated everolimus *plus* exemestane *versus* exemestane alone and the results are reported in 3 articles (BOLERO-2 trial). One phase III trial evaluated temsirolimus *plus* letrozole *versus* letrozole (HORIZON). One phase II trial evaluated sirolimus *plus* tamoxifen *versus* tamoxifen (Bhattacharyya trial). The included studies were published between 2011 and 2014. The total number of patients from all trials was 2147. The characteristics and efficacy results of the selected studies are reported in Table 1.

**Table 1:** Characteristics and efficacy results of the eligible studies

Trial	Author and year of publication	Phase	Design	Primary endpoints	Secondary endpoints	Number of enrolled patients
BOLERO-2	Yardley 2013 (PFS)	III	EXE+EVE	PFS	Safety, QoL, OS	485 EXE+EVE
	Piccart 2014 (OS)		*versus*			
	Rugo 2014 (AEs)		EXE+PBO			239 EXE+PBO
TAMRAD	Bachelot 2012	II	TMX+EVE	6-months CBR	TTP, OS, ORR, Toxicity	54 TMX+EVE
			*versus*			
			TMX			57 TMX
HORIZON	Wolff 2012	III	LETRO+TEMS	PFS	OS, Tumor Response, Clinical Benefit, TTP, TTF, Safety, QoL, Duration of Response	556 LETRO+TEMS
			*versus*			
			LETRO+PBO			556 LETRO+PBO
SIROLIMUS TRIAL	Bhattacharyya 2011	II	TMX+ SIR	TTP, ORR	Safety, Toxicity, Preliminary Pharmacoeconomic	200 TOTAL (PHASE II)
			*versus*			Analysis
			TMX			

Trial	Design	PFS/TTP (months)	HR (95%CI)	p	OS (months)	HR (95%CI)	p	ORR (%)	RR (95%CI)	p
BOLERO-2	EXE+EVE	7.8	0.45 (0.38-0.54)	<0.0001	31	0.89 (0.73-10)	NS (0.14)	12.6	0.88 (0.85-92)	<0.0001
	*versus*									
	EXE+PBO	3.2			26.6			1.7		
TAMRAD	TMX+EVE	8.6	0.54 (0.36-0.81)	0.0021	----	0.45(0.24-0.81)	0.007	14	1.35 (0.52-3.49)	NS
	*versus*									
	TMX	4.5						13		
HORIZON	LETRO+TEMS	8.9	0.90 (0.76-1.07)	NS (0.25)	----	0.89(0.65-1.23)	NS (0.5)	27	0.99 (0.82-1.21)	NS
	*versus*									
	LETRO+PBO	9						27		
SIROLIMUS TRIAL	TMX+ SIR	11	0.48 (0.25-0.93)	0.0028	----	----	----	40	----	----
	*versus*									
	TMX	3						4		

**EXE:** exemestane, **EVE**: everolimus, **PBO:** placebo, **TMX:** tamoxifene, **LETRO**: letrozole, **TEMS:** temsirolimus, **SIR**: sirolimus, **PFS:** progression free survival, **CBR:** clinical benefit rate, **TTP:** time to progression, **ORR**: objective response rate, **QoL**: Quality of Life, **OS:** overall survival, **TTF:** time to treatment failure, **NS:** not significant.

#### Everolimus

TAMRAD phase II randomized study investigated the efficacy and safety of everolimus 10 mg daily *plus* tamoxifen 20 mg daily *versus* tamoxifen alone in aromatase inhibitor (AI) resistant breast cancer patients. TTP (secondary endpoint) was 8.6 months in experimental arm *versus* 4.5 months in control arm (HR: 0.54; 95% CI 0.36-0.81; *p*=0.0021). A 55% reduction in risk of death was achieved in combination arm (HR: 0.45; 95% CI 0.24-0.81; *p exploratory* =0.007). ORR was 14% in tamoxifen *plus* everolimus and 13% in tamoxifen alone groups, respectively. Most common AEs in the combination group were stomatitis, fatigue, rash, diarrhea and anorexia [5].

In BOLERO-2 phase III randomized trial everolimus 10 mg daily *plus* exemestane 25 mg daily was compared to exemestane alone in postmenopausal women with hormone receptor positive HER2-negative advanced disease recurred or progressed after treatment with letrozole or anastrozole [6]. The median PFS (primary endpoint) was 7.8 months in combination therapy arm (485 patients) *versus* 3.2 months in control arm (239 patients) (HR: 0.45; 95% CI 0.38-0.54; *p*<0.0001) [7]. In post hoc analysis median post-progression survival in patients who had progressed at the time of the final PFS analysis was similar for both arms (20.8 months, 95% CI 17.3-23.3, *versus* 19.3 months, 95% CI 15.9-23.9). Final OS was 31 months (95% CI 28.0-34.6) in combination arm (482 patients) *versus* 26.6 months (95% CI 22.6-33.1) in HT alone (238 patients) (HR: 0.89; 95% CI 0.73-1.10; *p*=0.14) [8]. ORR was significantly higher for the combination therapy *versus* HT alone (12.6% *versus* 1.7%; *p*<0.0001) [7]. AEs mostly reported in experimental arm were pneumonitis, stomatitis, rash, dyspnea, fatigue [9]. The most common grade 3-4 AEs were reported in everolimus *plus* exemestane arm, with 22 deaths in combined arm and 4 deaths in placebo *plus* exemestane [8].

#### Temsirolimus

After a promising phase II trial on temsirolimus 30 mg daily for 5 days every 2 weeks and letrozole 2.5 mg daily *versus* letrozole alone, in postmenopausal women with recurrent or metastatic disease [10], the combination treatment was investigated in the phase III HORIZON, in postmenopausal hormone receptor positive women not treated with AI, with advanced or metastatic disease. The primary endpoint PFS resulted similar in both groups (HR: 0.90; 95% CI 0.76-1.07; *p*=0.25) and no differences in OS (HR 0.89; 95% CI 0.65-1.23) and in ORR (RR=0.99; 95% CI 0.82-1.21) were observed. Any grade and grade 3-4 AEs (stomatitis, diarrhea, rash, hyperglycemia) were mostly reported in the experimental arm [11].

#### Sirolimus

In a phase I-II trial patients who could not afford AI were randomized to tamoxifen 20 mg daily or tamoxifen *plus* sirolimus 2 mg daily and patients who had failed AI and/or tamoxifen were also randomized to the combination. In the phase II trial the primary endpoint TTP was improved by 3.3 months to 11.7 months adding sirolimus (HR: 0.43; 95% CI 0.25-0.92; *p*=0.0023) in patients progressed during treatment with prior AI or tamoxifen. In patients who had not received AI, sirolimus *plus* tamoxifen improved median TTP of 7 months compared to tamoxifen alone (HR 0.48; 95% CI 0.25-0.93; *p*=0.0028). The most common AEs in experimental arm were hyperglycemia, hypercholesterolemia, hypetriglicerydemia, stomatitis, rash and anorexia [12].

### Meta-analysis results

#### Efficacy

**Figure 2:**
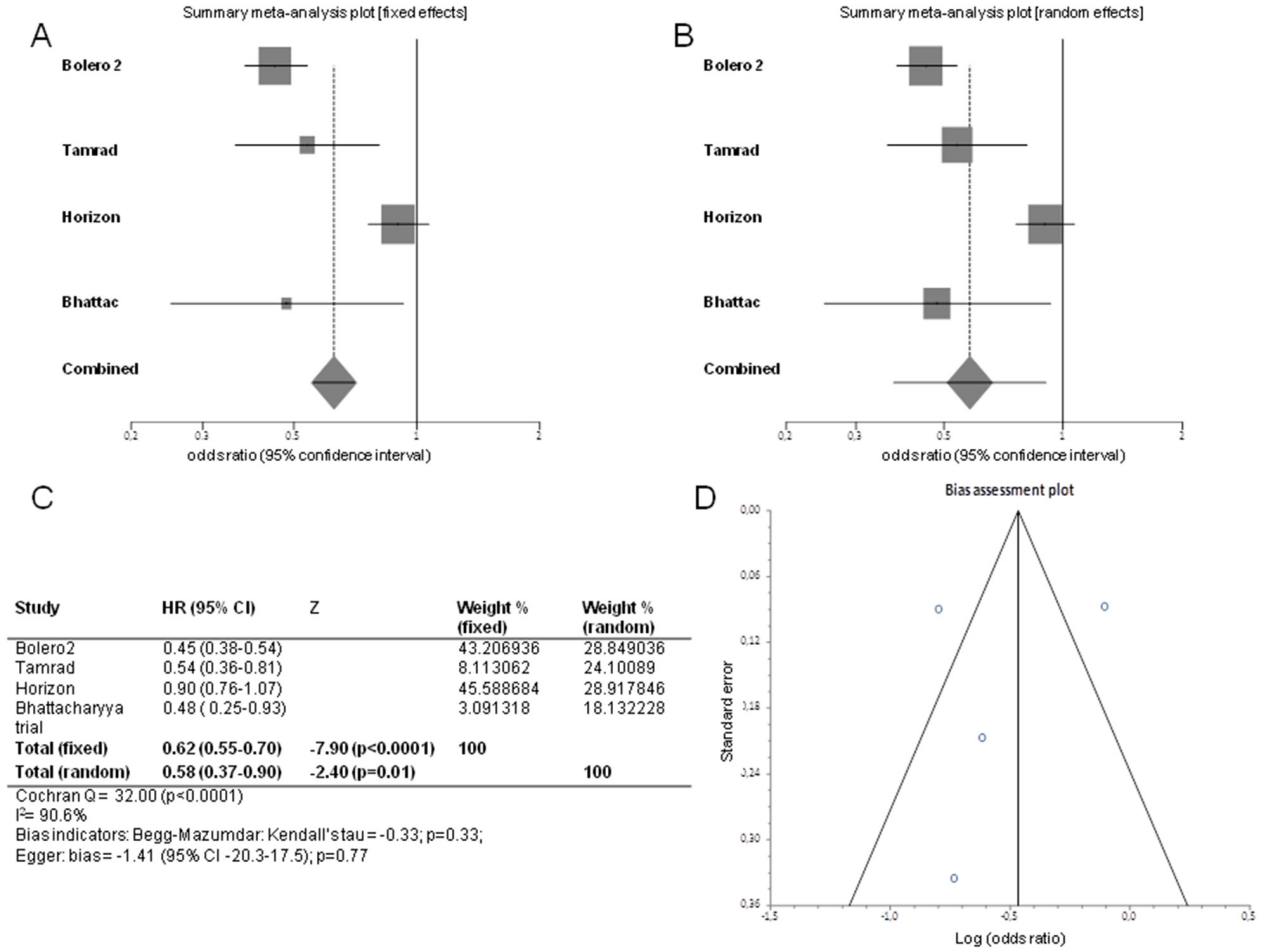
Forest plots of progression free survival/time to progression and Funnel plot for publication bias Pooled HR with 95% CI were generated with fixed and random effects models and the respective forest plots **A–B** are reported up in the figure. Cochran's Q and I^2^ index tests for detecting heterogeneity, Begg-Mazumdar and Egger tests for disclosing publication bias were performed **C.** A funnel plot **D** was drawn for checking bias.

**Figure 3:**
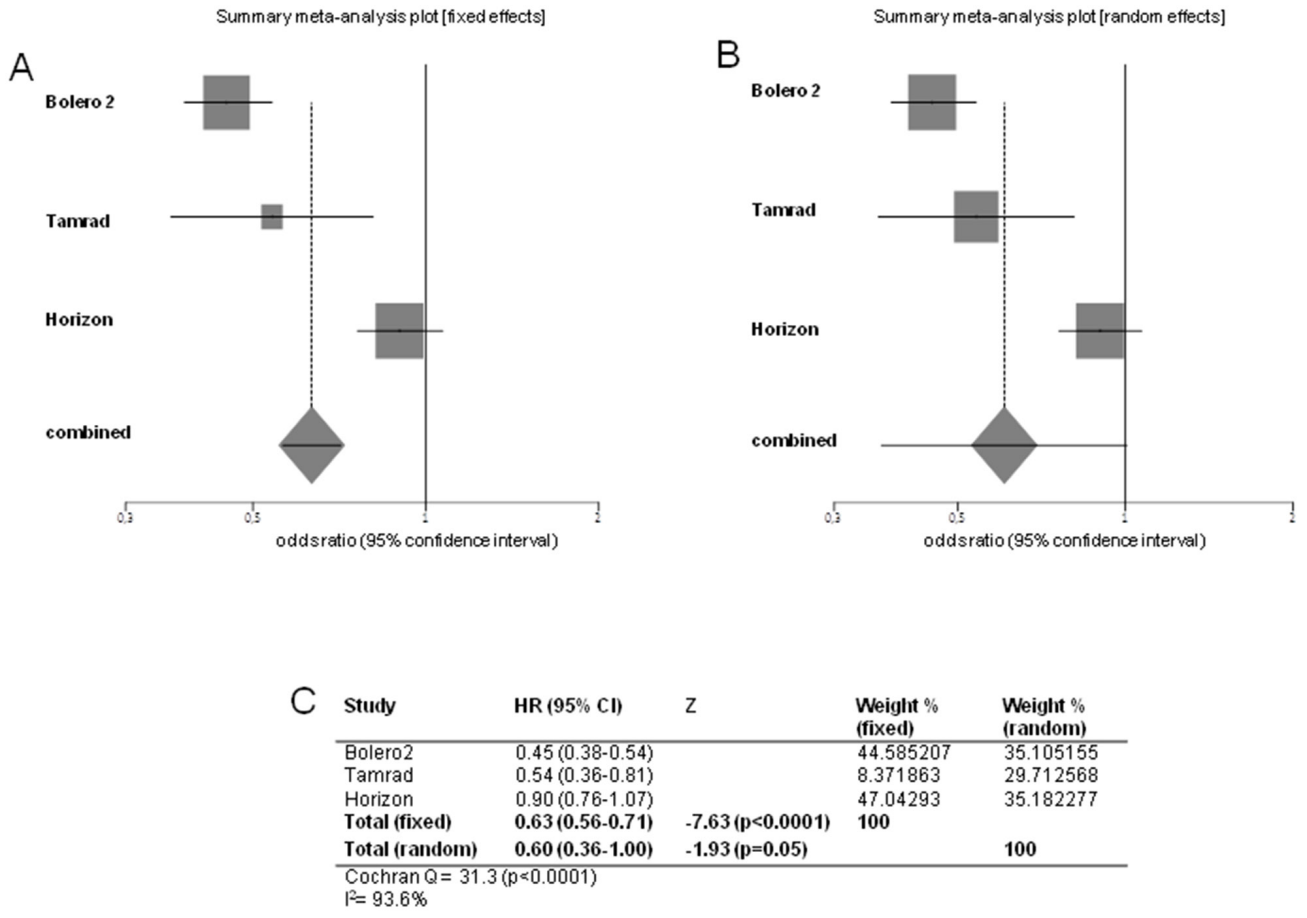
Forest plots of progression free survival/time to progression without Bhattacharyya trial Pooled HR with 95% CI were generated with fixed and random effects models and the respective forest plots **A–B** are reported. Cochran's Q and I^2^ index tests for detecting heterogeneity were performed **C.**

We evaluated PFS/TTP, OS and ORR to establish efficacy of mTOR-I+HT arm *versus* HT arm. Pooled HR for PFS/TTP, performed combining all the 4 trials, was 0.62 in favor of mTOR-I+HT arm (95% CI 0.55-0.70; *p*<0.0001) at fixed-effects model analysis. There was significant heterogeneity for PFS/TTP (Cochran's Q 32, *p*<0.0001, I^2^ index 90.6%). Pooled HR for PFS/TTP was 0.58 in favor of mTOR-I+HT arm (95% CI 0.37-0.90; *p*=0.01) at random-effects model analysis, too (Figure 2). The heterogeneity was still detected for PFS/TTP when Bhattacharyya trial was removed from the analysis (fixed-effects pooled HR 0.63; 95% CI 0.56-0.71; *p*<0.0001; Cochran's Q 31.3, *p*<0.0001, I^2^ index 93.6%; random-effects pooled HR 0.60; 95% CI 0.36-1.00; *p*=0.05) (Figure 3).

**Figure 4:**
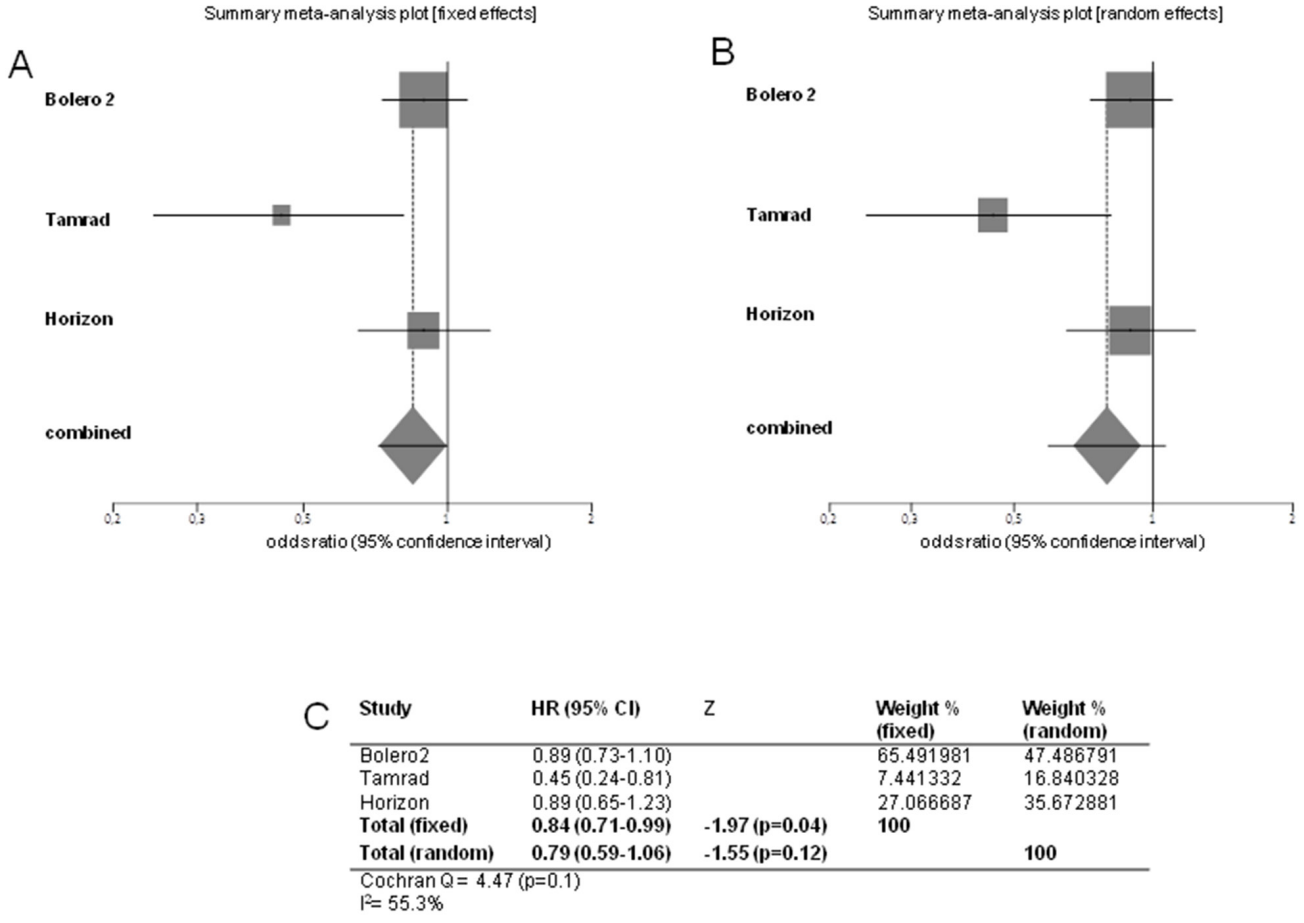
Forest plots of overall survival Pooled HR with 95% CI were generated with fixed and random effects models and the respective forest plots are reported **A–B.** Cochran's Q and I^2^ index tests for detecting heterogeneity were performed **C.**

Pooled HR for OS, performed excluding Bhattacharyya trial, was 0.84 in favor of the combination arm (95% CI 0.71-0.99; *p*=0.04). Heterogeneity was not significant (Cochran's Q 4.47, *p*=0.1, I^2^ index 55.3%) (Figure 4).

**Figure 5:**
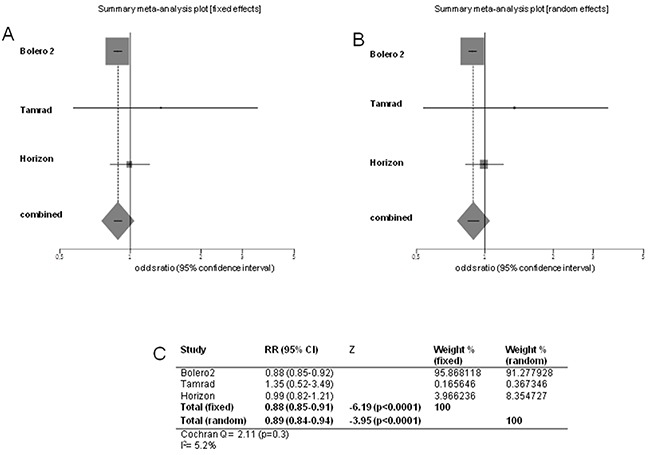
Forest plots of objective response rate Pooled RR with 95% CI were generated with fixed and random effects models and the respective forest plots are reported **A–B.** Cochran's Q and I^2^ index tests for detecting heterogeneity were performed **C.**

Pooled RR for ORR, performed excluding Bhattacharyya trial, was 0.88 in favor of experimental arm (95% CI 0.85-0.91; *p*<0.0001). Heterogeneity was not significant (Cochran's Q 2.11, *p*=0.3, I^2^ index 5.2%) (Figure 5).

#### Safety

Data for each AE were extracted in those trials reporting them consistently. The pooled RR for AEs was performed using the fixed-effects model analysis. A random-effects model analysis was added for a more conservative estimate in the presence of heterogeneity. The I^2^ index has been evaluated in addition to Cochran's Q. Any grade diarrhea, pneumonitis, infection and dyspnea resulted significantly more frequent in the mTOR-I+HT arm and the heterogeneity did not show significant difference at Cochran's Q test. However for diarrhea the I^2^ index was high (73.4%), while for pneumonitis, infection and dyspnea the I^2^ index was medium (39.7%), medium (55.3%) and low (0%), respectively. Any grade asthenia, fatigue, stomatitis and rash were significantly more frequent in the experimental arm, but the heterogeneity was significant at Cochran's Q test and it was confirmed by I^2^ index. Asthenia and fatigue did not show statistical differences between arms at random-effects model analysis. Hyperglycemia did not show differences between arms, too, but significant heterogeneity resulted at Cochran's Q test and at I^2^ index. Grade 3-4 asthenia, fatigue, diarrhea, pneumonitis, rash and dyspnea significantly resulted more frequent in the mTOR-I+HT arm and the heterogeneity did not show significant difference at Cochran's Q test (I^2^ index: 0% for asthenia, diarrhea, pneumonitis and dyspnea, 6.3% for fatigue, 64% for rash, respectively). Grade 3-4 infection resulted similar in both arms and the heterogeneity did not show significant difference at Cochran's Q test, with a low I^2^ index (0%). Grade 3-4 stomatitis and hyperglycemia resulted significantly higher in combination arm at fixed-effects model, but not at random-effects model analysis, with a significant heterogeneity at Cochran's Q test and an I^2^ index of 68.4% for stomatitis and 84.8% for hyperglycemia, respectively (Table 2).

**Table 2:** Meta-analysis results: safety

Meta-analysis of any grade adverse events.								
	Fixed-effects RR (95% CI)	Z	P	Heterogeneity Cochran's Q (P)	I^2^ INDEX (%)	Random-effects RR (95%CI)	Z	P
Asthenia	0.989	−2.48	0.01	4.90	79.6	0.961	−1.06	0.28
	(0.981-0.997)			(0.02)		(0.895-1.033)		
Fatigue	0.97	−2.63	0.008	4.06	75.4	0.80	−0.85	0.39
	(0.96-0.99)			(0.04)		(0.48-1.32)		
Diarrhea	0.85	−6.86	<0.0001	3.76	73.4	0.78	−2.10	0.03
	(0.81-0.89)			(0.05)		(0.62-0.98)		
Stomatitis	0.95	−6.33	<0.0001	37.28	94.6	0.87	−2.49	0.01
	(0.94-0.96)			(<0.0001)		(0.78-0.97)		
Pneumonitis	0.93	−5.97	<0.0001	1.65	39.7	0.92	−2.21	0.02
	(0.91-0.95)			(0.19)		(0.85-0.99)		
Hyperglycemia	0.996	−1.95	0.05	21.16	95.3	0.95	−1.02	0.30
	(0.992-1.00)			(<0.0001)		(0.87-1.04)		
Infection	0.95	−2.44	0.01	2.23	55.3	0.91	−1.12	0.25
	(0.92-0.99)			(0.13)		(0.77-1.07)		
Rash	0.97	−5.49	<0.0001	41.90	95.2	0.86	−2.30	0.02
	(0.96-0.98)			(<0.0001)		(0.77-0.97)		
Dyspnea	0.96	−4.22	<0.0001	0.17	0	0.96	−4.22	<0.0001
	(0.95-0.98)			(0.67)		(0.95-0.98)		

Meta-analysis of grade 3-4 adverse events.								
Asthenia	0.994	−1.97	0.04	0.22	0	0.994	−1.97	0.04
	(0.989-0.999)			(0.63)		(0.989-0.999)		
Fatigue	0.991	−2.12	0.03	1.06	6.3	0.99	−0.64	0.52
	(0.983-0.999)			(0.30)		(0.97-1.01)		
Diarrhea	0.98	−2.79	0.005	0	0	0.98	−2.79	0.005
	(0.96-0.99)			(>0.99)		(0.96-0.99)		
Stomatitis	0.991	−2.22	0.02	6.33	68.4	0.98	−1.27	0.20
	(0.983-0.998)			(0.04)		(0.97-1.00)		
Pneumonitis	0.98	−2.43	0.01	0.80	0	0.98	−2.43	0.01
	(0.96-0.99)			(0.37)		(0.96-0.99)		
Hyperglycemia	0.996	−1.99	0.04	6.56	84.8	0.98	−1.08	0.27
	(0.992-0.999)			(0.01)		(0.95-1.01)		
Infection	0.98	−1.69	0.09	0.16	0	0.98	−1.69	0.09
	(0.97-1.001)			(0.68)		(0.97-1.001)		
Rash	0.988	−3.95	<0.0001	5.55	64	0.98	−2.26	0.02
	(0.982-0.994)			(0.06)		(0.97-0.99)		
Dyspnea	0.98	−3.90	<0.0001	0.77	0	0.98	−3.90	<0.0001
	(0.97-0.99)			(0.37)		(0.97-0.99)		

#### Publication bias

A funnel plot was drawn for PFS/TTP and no asymmetry was detected. P value was 0.33 at Begg-Mazumdar test and 0.77 at Egger test, respectively, providing statistical evidence of funnel plot symmetry and then of the absence of publication bias (*p*>0.05). These tests cannot perform when >3 trials data are not available. So we could not check meta-analysis biases by the other outcomes (Figure 2).

## DISCUSSION

In the light of the most recent advances, the standard of care of metastatic luminal breast cancer is nowadays represented by HT in those cases with favorable prognostic features [13].

mTOR is a serine/threonine protein kinase downstream PI3K/Akt pathway, which controls cell growth, proliferation, survival, metabolism and angiogenesis and is involved in cancer development [14]. Rapamycin (sirolimus) and its analogs CCI-779 (temsirolimus), RAD001 (everolimus) and AP23573 inhibit mTOR activation, that is often involved in mechanisms of anticancer drug resistance [15]. On this basis, a potential strategy to antagonize cellular HT escape is to target mTOR or its functionally related signalling proteins, such as PI3K, PTEN, AKT [16].

In preclinical studies, temsirolimus inhibited mTOR and restored sensitivity to tamoxifen primarily through induction of apoptosis, suggesting that AKT-induced resistance to tamoxifen may be mediated by escape from cell death [17]. Preliminary studies showed that everolimus reversed AKT-mediated cell resistance and restored responsiveness to letrozole or fulvestrant [18]. On these bases, there is a strong rationale for combination of anti-estrogen drugs with rapamycin analogs. However, tumors with high PI3K-AKT-mTOR activity are heterogeneous in response and novel biomarkers are required to identify breast cancer subtypes that could benefit from the combination [19]. The results of current clinical trials appear, as first impact, to confirm the preclinical findings. The addition of a mTOR-I to HT resulted in improved TTP in TAMRAD trial (HR: 0.54; 95% CI 0.36-0.81) [5], improved PFS in Bhattacharyya trial (HR: 0.48; 95% CI 0.25-0.93; *p=*0.0028) [12], improved PFS in BOLERO-2 trial (HR 0.45; 95% CI 0.38-0.54; *p*<0.0001) [7], while same results were not demonstrated for temsirolimus in the large HORIZON trial population, that showed similar PFS in both arms (HR 0.90; 95% CI 0.76-1.07; *p*=0.25). The results of HORIZON study can be explained by exclusion of patients pretreated with AI, who could not be considered HT-resistant [11].

The benefits derived from individual trials have been significantly maintained in the whole meta-analysis population (pooled HR for PFS/TTP: 0.62; 95% CI 0.55-0.70; *p*<0.0001). However, study heterogeneity for PFS/TTP was found and could be also demonstrated when Bhattacharyya trial was removed from the analysis.

For the OS endpoint, TAMRAD trial moved the balance in favor of combination (HR 0.45; 95% CI 0.24-0.81; *p*=0.007), taking into account that the other trials were not significant on this endpoint [5, 8, 11, 12]. In meta-analysis pooled OS was significantly prolonged in the mTOR-I+HT arm, with a 16% reduction of risk of death (*p*=0.04); data were homogeneus. Bhattacharyya trial did not report OS data.

ORR was higher in experimental arm in BOLERO-2 trial (12.6% *versus* 1.7%; *p*<0.0001) [7], while it was similar in both arms in TAMRAD (14% *versus* 13%) [5] and HORIZON (27% *versus* 27%) [11]. Pooled RR for ORR, performed without Bhattacharyya trial, was 0.88 (*p*<0.0001) in favor of the experimental arm. Heterogeneity was not significant.

AEs, in particular those of grade 3-4, were mostly increased in mTOR-I+HT arm both in single trials [5, 8, 9, 11, 12] and in meta-analysis pooled data, even for cases of rare toxicity-related deaths. The most common AEs that led to dose interruptions/reductions, such as fatigue, pneumonitis and dyspnea [5, 8, 9, 11, 12], were also referred to the combination arm. The sirolimus study was not included because data on toxicity were not available.

All together, these results suggest that combination therapy with mTOR-I improves the efficacy of HT in metastatic luminal breast cancer patients and impacts on survival. The reported heterogeneity relies on various factors, for example heterogeneous study populations (HT naïve in HORIZON, in failure after previous HT in the other studies) and imbalance in post-study treatment lines. BOLERO-2 trial and HORIZON trial sample sizes were weighted on PFS primary endpoint and not on OS, while TAMRAD trial sample size was based on clinical benefit rate as primary endpoint. In TAMRAD trial and in Bhattacharyya trial only TTP was selected as endpoint. Finally, in Bhattacharyya trial, primary endpoints were ORR and TTP. Many potential limitations can affect our results, because differences in the design, patients characteristics, methodological quality and execution of the single primary studies could not be overcame. An additional important point is that, even if results were obtained from randomized clinical trials, we did not handle individual patients data, but whole populations data were extracted from the available reports. Moreover not all outcomes were reported in all studies and our work was retrospective. Risks of selection or publication biases, due to selection of studies that report dramatic effects and to exclusion of studies that report smaller effect sizes, have not been shown in our meta-analysis, except for PFS/TTP, where no funnel plot asymmetry was detected. Furthermore, as previously described, we repeated the analysis for PFS/TTP excluding one of the original studies (Bhattacharyya trial) to determine if the overall conclusions have been influenced by study selection.

In the light of our results, we can conclude that mTOR-I offer a benefit as a class-effect, even though the differences among drugs investigated in the individual trials and among population characteristics should not be overlooked.

The use of mTOR-I offers a benefit that must be weighted in the clinical scenario of metastatic luminal breast cancer. In palliative setting, the gain of few months in PFS/TTP, showed in our work, should be balanced with the patient quality of life, which rather could be affected by side effects of mTOR-I treatment. In particular, in asymptomatic luminal patients with good prognosis, the combination treatment should be comparable to the HT alone in terms of toxicity to ensure the maintenance of good clinical conditions. Cost-effectiveness analysis demonstrated conflicting results on everolimus *plus* exemestane compared to exemestane alone in BOLERO-2 [20]. Long-term results and analysis of post-marketing studies are indeed needed to finally address this important issue. A further point is the inclusion of mTOR-I in the therapeutic algorithm for patient continuum of care. At present mTOR-I have been investigated in neoadjuvant setting, with limited benefits [21], while new studies are ongoing in the adjuvant setting [22–23]. In metastatic disease, the trials included in our meta-analysis allocated the combination treatment in HT naïve or in patients who failed HT. Evidence in favor of combined HT *plus* mTOR-I rather than chemotherapy with or without biological agents, such as bevacizumab in HER-2 negative breast cancer, in first line or in subsequent lines, is not still available. This comparison is indeed very difficult, due to selection bias in favor of chemotherapy for patients with more aggressive disease. However, although chemotherapy is the mainstay in patients at risk of visceral crisis, BOLERO-2 subgroup analysis showed that patients with visceral metastasis can indeed benefit from everolimus and exemestane combination [24].

Finally, the role of novel agents that can potentiate mTOR blockade, is under investigation. The combination of PI3K and CDK4/6 inhibitors demonstrated promising data on apoptosis induction, due to sensitization of ER-positive cells to CDK4/6 inhibition by suppressing cyclin D1 expression [25]. Targeting the PI3K pathway, such as by dual inhibitors of PI3K and mTOR, is another strategy presently under investigation [26].

We think that our study provides an important proof-of-concept that interference with mTOR is a crucial biologic mechanism regulating hormone sensitivity in luminal breast cancer, that must be weighted in the clinical scenario and further efforts are necessary in order to correctly allocate these agents in the patient *continuum* of care.

## PATIENTS AND METHODS

We provided to search trials on public databases and the analysis was performed in accordance with the Preferred Reporting Items for Systematic Reviews and Meta-analyses (PRISMA) guidelines (PRISMA 2009 checklist) [27]. No study involving human participants and requiring ethics committee approval in accordance to the Declaration of Helsinki and its subsequent revisions was conducted during our investigation.

### Eligibility criteria

#### Inclusion criteria

The studies concerned the diagnosis of metastatic luminal HER2-negative breast cancer. The trials might be randomized, prospective and controlled. We considered congress abstracts if containing sufficient information about the study design, the patient characteristics, outcomes and toxicity. Patients in the experimental arm received mTOR-I *plus* HT. Patients in the control arm received only HT.

#### Exclusion criteria

No comparative studies, no randomized clinical trials, studies that not involved our target drugs, studies with no comparable endpoints, other than oral administration and languages other than English were excluded.

### Information sourches and search strategy

Public databases (PubMed, Embase, Central Registry of Controlled Trials of the Cochrane Library) full texts and abstracts were interrogated for the 2005-2015 time frame. Google academic search (including also meeting abstracts) was performed to track relevant references, too. The search included the following keywords: “breast” AND (“cancer” OR “cancers” OR “carcinoma” OR “carcinomas”) AND “phase II” OR “phase III” AND “mTOR” AND (“inhibitor” OR “everolimus” OR “RAD001” OR “rapamycin” OR “sirolimus” OR “PI-103” OR “temsirolimus” OR “torisel”).

### Study selection and data collection process

The studies were examined independently by two investigators in order to select eligible studies (MSR, TG). Numerous selected variables were extracted and evaluated, such as the number of enrolled patients, year of publication, the treatment program and activity and efficacy endpoints. The data concerning the occurrence of toxicity were obtained from the safety profile of each study. Any discrepancy was resolved by an arbitrator (PT). We considered all patients for PFS/TTP, OS, ORR and toxicity, with any grade and grade 3-4 AEs.

### Summary measures and statistical analysis

HR with their corresponding 95% CI for PFS/TTP and OS, RR for ORR and for any grade and grade 3-4 AEs were compared in the two groups: mTOR-I+HT *versus* HT alone. The meta-analysis was performed with an alpha error of 0.05 and the *p* value <0.05 was considered to be significant. A fixed-effects model was chosen and analysis by random-effects model was added to generate a more conservative estimate when the heterogeneity at Cochran's Q test showed significant difference (*p*<0.05). In addition to Cochran's Q, the I^2^ index has been performed to quantify the degree of heterogeneity and to give a better measure of the inter-trials consistency. For higher values of I^2^ index, heterogeneity is more enhanced (I^2^ index of 25%, 50% and 75% corresponds to low, medium and high heterogeneity, respectively). Begg and Egger tests were performed to evaluate potential publication biases and they were considered significant when the *p-*value resulted <0.05. Forest plots are reported to display the meta-analysis results and a funnel plot was drawn to detect biases when we could evaluate >3 trials (pooled HR for PFS/TTP). We used Stats Direct 3 software for statistical analysis.
